# Platelet and Cancer-Cell Interactions Modulate Cancer-Associated Thrombosis Risk in Different Cancer Types

**DOI:** 10.3390/cancers14030730

**Published:** 2022-01-30

**Authors:** Ana-Luisa Palacios-Acedo, Mélanie Langiu, Lydie Crescence, Diane Mège, Christophe Dubois, Laurence Panicot-Dubois

**Affiliations:** 1 Aix Marseille University, INSERM 1263 (Institut National de la Santé et de la Recherche), INRAE 1260 (Institut National de la Recherche Agronomique et de l’Environnement), C2VN (Center for CardioVascular and Nutrition Research), 13885 Marseille, France; ana-luisa.palacios-acedo@univ-amu.fr (A.-L.P.-A.); melanie.langiu@univ-amu.fr (M.L.); lydie.crescence@univ-amu.fr (L.C.); diane.mege@univ-amu.fr (D.M.); laurence.panicot-dubois@univ-amu.fr (L.P.-D.); 2 Marseille University, PIVMI (Plateforme d’Imagerie Vasculaire et de Microscopie Intravitale), C2VN (Center for CardioVascular and Nutrition Research), 13385 Marseille, France; 3 Department of Digestive Surgery, La Timone University Hospital, 13005 Marseille, France

**Keywords:** cancer, thrombosis, platelets, risk, cancer-associated thrombosis, Trousseau’s syndrome, pancreatic, oral, colorectal

## Abstract

**Simple Summary:**

Cancer-associated thrombosis is the first cause of death in cancer after cancer-progression itself; however, thrombotic risk is not the same in all cancer types. Here, we exemplified cancer-cell interactions with platelets in tumor microenvironments and their effect on overall thrombotic risk. We chose three distinct cancer types with varying thrombotic risks: oral cancer (low risk), colorectal cancer (medium risk) and pancreatic cancer (high risk). Understanding these interactions and their consequences is crucial for deepening researcher’s and clinician’s knowledge of cancer-associated thrombosis and finding innovative biomarkers and therapeutic targets.

**Abstract:**

The first cause of death in cancer patients, after tumoral progression itself, is thrombo-embolic disease. This cancer-associated hypercoagulability state is known as Trousseau’s syndrome, and the risk for developing thrombotic events differs according to cancer type and stage, as well as within patients. Massive platelet activation by tumor cells is the key mediator of thrombus formation in Trousseau’s syndrome. In this literature review, we aimed to compare the interactions between cancer cells and platelets in three different cancer types, with low, medium and high thrombotic risk. We chose oral squamous cell carcinoma for the low-thrombotic-risk, colorectal adenocarcinoma for the medium-thrombotic-risk, and pancreatic carcinoma for the high-thrombotic-risk cancer type. We showcase that understanding these interactions is of the highest importance to find new biomarkers and therapeutic targets for cancer-associated thrombosis.

## 1. Introduction

The connection between thrombosis and cancer has been known for nearly 150 years since Dr. Armand Trousseau described migratory thrombophlebitis as a diagnostic element for visceral cancer [1]. Today, we know that 20 to 30% of first-time diagnoses of venous thrombotic events are cancer-related, and they portend poor prognosis with low survival odds for those that develop them [2,3]. Indeed, thrombotic events are the second cause of death in oncologic patients, only after disease progression itself. Cancer patients have an increased incidence of both venous (4–20%) and arterial (2–5%) thrombotic events [4,5]. However, the incidence of cancer-associated thrombosis (CAT) can vary depending on tumor type, stage, and administered treatment, and often increases over time [3]. This increase in thrombotic events is most likely multifactorial, due to disease progression as well as treatments (many commonly used therapeutic agents are known to increase venous thromboembolism risk) and the fact that cancer treatments are associated with longer survival rates [6,7].

The most important modulator of thrombotic risk in cancer patients seems to be the tumor site and burden. The primary sites most linked to increased CAT include pancreas, lung, lymphoma, stomach, and testicular cancer [8]. Primary brain tumor and hematologic malignancies are also at high risk of venous thromboembolism (VTE) development [9]. The stage of the cancer also positively correlates with thrombotic risk in almost all cancer types [7].

Incidentally, the genetic and mutational status of tumors can also have an impact on CAT development. Indeed, *ALK* and *ROS-1* rearrangements in non-small-cell-lung cancer as well as *KRAS* mutations in colorectal and lung cancer are known to confer a higher thrombotic risk [8,10,11]. In another study, Dunbar and collaborators did a large-scale analysis to determine if tumor-specific genomic events could be related to CAT risk and found that somatic mutations of genes were associated with heightened malignant aggressiveness: *STK11*, *KRAS*, *CTNNB1*, *KEAP1*, *CDKN2B*, and *MET* were all associated with increased risk in patients with solid tumors [12]. These genes all have different functions and effects: KRAS is a tumor-specific mutation that promotes the initiation and progression of cancers, and *STK11* is a tumor suppressor that loses function after mutations [13,14]. *CTNNB* mutations cause the aberrant activation of the β-catenin gene, while KEAP1 mutations, which are common in lung cancer, produce an improved oxidative stress response in cancer cells and are associated with aggressive disease and shorter overall survivals [15,16]. *CDKN2B* encodes for cyclin-dependent kinase 6 (CDK6), which regulates the cell cycle; its mutation in the cancer setting is associated with uncontrolled tumor growth [17]. On the other hand, MET mutations have been associated with a hypercoagulable state in animal models of cancer [18]. In another study, it was shown that BRCA2 mutations are related to increased plasma levels of P-selectin, PF4, and fibrinogen, which seems to suggest that coagulation may be heightened in this case [19]. On the other hand, IDH1/2 mutations are correlated with lower thromboembolic risk in patients with glioblastomas [20,21].

The intrinsic characteristics of the tumor itself are mostly responsible for the increase in coagulation risk in oncological patients. However, this risk can be modified and even exacerbated by the specific characteristics of patients [7,8]. For example, being female, of more than 65 years of age, of black ethnicity, and with elevated body-mass index can significantly increase the risk of developing blood clots [8,9]. The presence of comorbidities such as renal, cardiovascular, and lung disease, as well as a precedent VTE event, also increases the risk of developing blood clots [8,9].

Traditionally, the main reason for the cancer-associated hypercoagulable state known as Trousseau’s syndrome is that tumor cells can interact with and activate platelets [22]. However recent publications show that there are many cell types and molecules involved, creating a web of pro-thrombotic interactions around the tumor [5]. Different cancer-type specific pathways of thrombosis have been proposed to explain the risk difference between cancer types and risk-assessment scores [5]. These extensive interactions are why scores such as the Khorana and PROTECT scores use parameters such as cancer site as well as circulating blood cell counts (thrombocytosis, leukocytosis) before the start of treatment, as well as treatment type, to stratify risk [5,23].

The interaction between cancer cells with platelets and neutrophils—and their releasates—can also impact and promote different paths for increased thrombotic risk [22,24,25,26,27,28]. Indeed, when platelets degranulate they release pro-inflammatory molecules that act as chemo-attractants for neutrophils in the tumoral microenvironment [27]. Neutrophils increase the local inflammation and can undergo a switch to a protumoral “N2” phenotype [29]. Neutrophils enhance cancer-associated thrombosis by expressing procoagulant molecules such as P-selectin and tissue factor on their surface or by secreting neutrophil extracellular traps that enhance the thrombotic risk by trapping platelets and coagulation factors [30,31,32,33,34,35]. In this article, we will focus specifically on the interactions between cancer cells and platelets in the context of CAT.

## 2. Platelet and Cancer-Cell Interactions

Platelets are anucleate cells produced by megakaryocytes in the bone marrow; they have a discoid shape and measure 2–5 µm in diameter [22,36]. When activated by different agonists, most notably thrombin, ADP, von Willebrand factor (vWF), and collagen, platelets change shape and produce multiple filopodia [22,36,37]. Platelets have specific receptors for their agonist molecules, for example, thrombin can bind to GPIb-IX-V and the PAR receptors (with PAR_1_ being more sensitive than PAR_4_ in human), and ADP can bind to P2RY1 and P2RY12, who, respectively, initiate and amplify platelet degranulation and aggregation [22]. Von Willebrand factor exposed on activated endothelial cells or on the subendothelium matrix will interact with platelet GPIbα and αIIbβIII integrin to cross-link activated platelets during thrombus formation [22,38]. Exposed collagen can also bind to platelets through the integrin αIIβI and GPVI receptor and contribute to platelet activation and aggregation [22,37].

**Figure 1:**
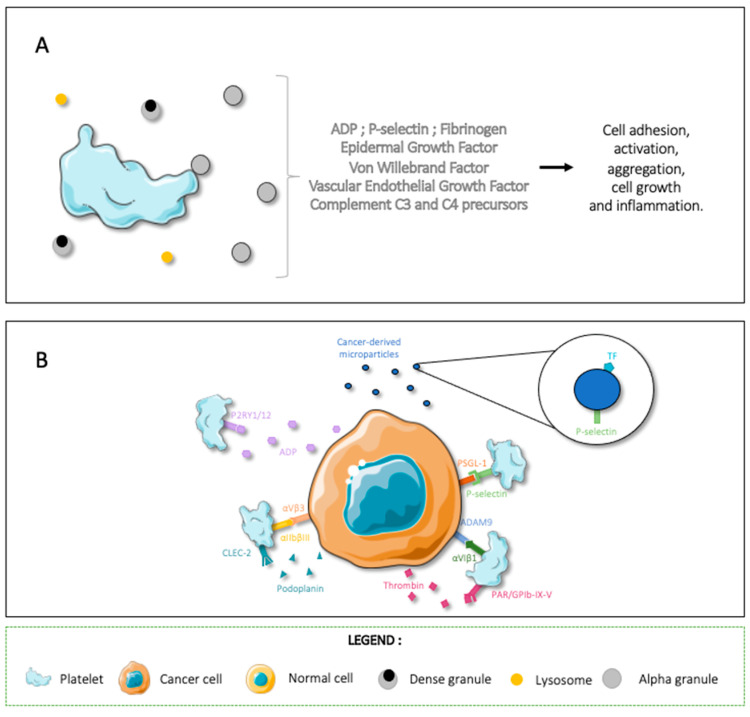
Schematic representation showing how the interaction of platelets with cancer cells can induce cancer-associated thrombosis. (**A**) Platelets have three distinct types of granules: alpha, dense, and lysosomal. α-granules are the most present and contain both membrane-associated and soluble proteins that promote cell adhesion, activation, aggregation, cell growth, and inflammation through interactions with other cell types. (**B**) Cancer cells can express platelet agonists, intermediary molecules, and microvesicles to activate platelets and induce TCIPA. Figure created using Servier Medical Art available at http://smart.servier.com/, accessed on 15 December 2021.

Activated platelets will both degranulate and express activation and adhesion molecules on their surface [2,22,36]. Platelets have three distinct types of granules: alpha, dense, and lysosomal. α-granules are the most present and contain both membrane-associated and soluble proteins such as ADP, P-selectin, fibrinogen, epidermal growth factor, vWF, vascular endothelial growth factor (VEGF), and complement C3 and C4 precursors (Figure 1A) [22]. When granule contents are released, the contents of the granules are either released into the microenvironment or upcycled to the platelet membrane [22,39,40]. The granule components promote cell adhesion, activation, and aggregation; they can also promote cell growth and inflammation through interactions with other cell types [22,39,40].

Tumor and platelet cell interactions can happen directly or indirectly through the release of platelet agonists or intermediary molecules and microvesicles (MVs) [22]. The most common effect of these interaction is tumor-cell-induced platelet aggregation (TCIPA) [22]. TCIPA is correlated with higher aggressiveness and metastatic potential due to increased survival of tumor cells in circulation [22,41]. In order to activate platelets in the tumoral microenvironment and induce TCIPA, cancer cells can express platelet agonists on their membrane. This platelet activation in the tumoral microenvironment contributes to cancer progression [22]. Activated platelets “hide” metastatic cells from the immune system and protect them from shear forces in circulation [22,36,42]. Activated platelets can also degranulate in the tumoral microenvironment and provide pro-growth and pro-angiogenetic signals to the surrounding stroma [22,43,44]. As an example, platelet receptors αIIbβIII and αVIβ1 bind, respectively, to cancer-cell αVβ3 and ADAM9, and tumoral cells can interact with platelet P-selectin through PSGL-1 [22,45]. Cancer-cells can also express molecules such as podoplanin and thrombin, which interact with platelet CLEC-2 and PAR_1/2_ receptors to activate and aggregate platelets [22,44,46,47,48]. Evidently, these activated platelets degranulate in the tumoral microenvironment and amplify the TCIPA loop.

Interestingly tumor-cells themselves can also secrete platelet agonists into their microenvironment. These secreted molecules contribute to the local TCIPA as well as recruit immune cells such as tumor-associated neutrophils and macrophages into the tumoral stroma [49,50]. Among these pro-platelet molecules, we have tumor-cell ADP secretion, which enhances platelet activation through P2RY1 and 12; in turn, platelet releasates attract immune cells and perpetuate local inflammation [22,51].

Cancer-cells are also known for releasing large numbers of MVs into the tumoral microenvironment [22]. MVs are plasma membrane vesicles produced by activated or apoptotic cells as well as resting cancer cells. These vesicles contain the parent cell’s membrane markers and may have mRNA, proteins, and some organelles in their cytoplasm [52,53,54]. MVs are vectors of information between different cell types and tissues. Interestingly, cancer-derived MVs have exposed negative phospholipids that support platelet function, thus supporting the development of CAT [22,47]. In some cancers, such as pancreatic adenocarcinoma, the tumor-derived MVs express procoagulant molecules such as tissue factor (TF) and podoplanin and adhesion molecules such as P-selectin, allowing them to interact with platelets and other cell types to promote tumor growth and metastasis [22,55,56]. As information vectors, they also have mRNA, which can alter the transcriptome of targeted cells [54].

The interactions between tumor cells and platelets can also induce changes in the protein profile of circulating platelets, emphasizing the bi-directional communication between these cell types [40,57,58]. Ercan and colleagues have shown that the proteome in platelets from patients with lung cancer was significantly different to that of matched healthy samples [58]. They found specifically an over-expression of endoplasmic reticulum proteins: careticulin, endoplasmic reticulum chaperone BiP, and protein disulfide-isomerase [58]. These altered protein levels were associated with increased platelet degranulation and upregulation of protein processing in the endoplasmic reticulum. Authors also found elevated levels of a coagulation factor XIII fragment named F13A1, which suggest higher activation rate of fXII and thus coagulation cascade activation in lung cancer patients [58,59]. When they compared platelet proteome of brain cancer patients with healthy samples, they found that brain cancer patients did not have altered proteomes such as in lung cancer, showing that each cancer type has unique interactions and effects on platelets and vice versa [58].

As the evidence shows, platelets and cancer cells are in constant interaction, both in the tumoral microenvironment and in circulation (Figure 1B). However, the final effect these interactions have depends on the cancer type. In this review article, we will expose three cancer types with different thrombotic risks and showcase the cancer–platelet interactions within each.

## 3. Oral Cancer

Head-and-neck cancer (HNC) represents approximately 2% of all cancers worldwide and has a 5-year survival rate of 60% [60]. The most common localizations in the oral cavity are oral cavity (lips, tongue, and mucosa), pharyngeal, and laryngeal, and 90% are squamous cell carcinoma [61].

Despite the fact that HNC is between the 8th and 10th most common cancer in the world, there is surprisingly little information available about the risk of developing thrombotic events in this disease [60,61,62]. In a worrisome manner, the incidence of oral cavity and pharyngeal cancer is on the rise at a rate of 0.6% per year in the last decade alone [63]. Interestingly, the known risk factors for developing this disease are similar to those associated with heightened thrombotic risk; certainly, risk factors such as tobacco use and an age over 50 years are common to both diseases [61,64]. In developed countries, the incidence of HNC associated with Human Papilloma Virus is on the rise, particularly in younger patients, underlining the need to better understand this cancer and its possible complications [61,65].

**Figure 2:**
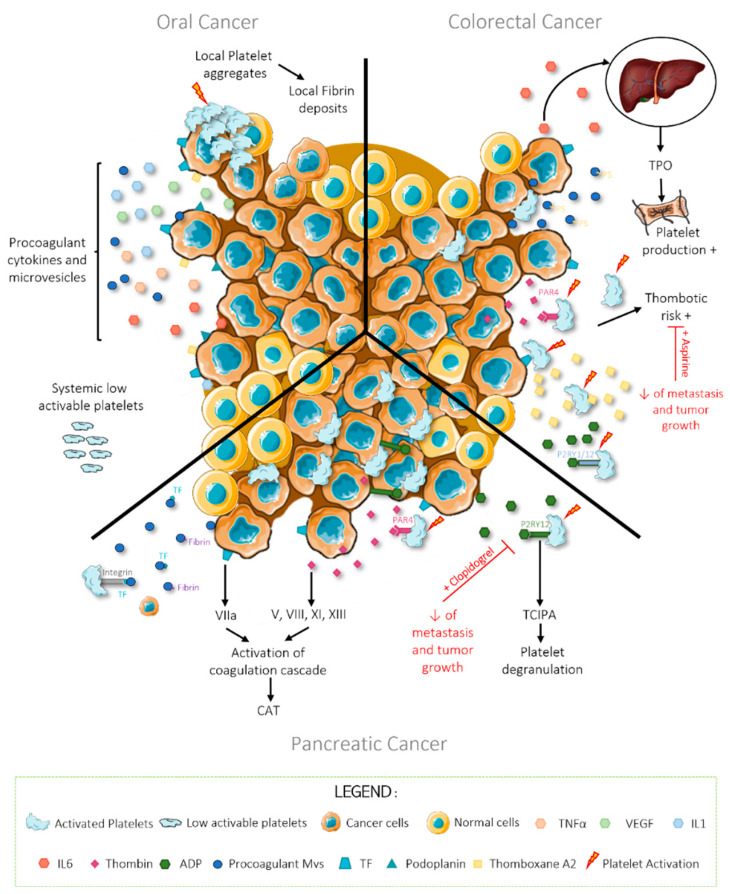
Comparison of cancer-cell–platelet interactions in three different cancer types with low (oral cancer), medium (colorectal cancer), and high (pancreatic cancer) thrombotic risk. Figure created using Servier Medical Art available at http://smart.servier.com, accessed on 15 December 2021.

It is generally accepted that head and neck cancers have low thrombotic risk, and a clinical study review performed by P. Haen and collaborators in 2019 confirms this [61]. However, HNC and, more specifically, oral squamous cell carcinoma (OSCC) has multiple characteristics of cancers associated with high thrombotic risk, for example, modified thrombosis and fibrinolysis mechanisms, strong expression of procoagulant factors, and production of procoagulant cytokines and of procoagulant microparticles [62,66,67,68,69,70,71]. Indeed, OSCC cells can express TF, podoplanin, and thromboxane A2 (TXA2) and secrete TNFα, interleukins IL1 and IL6, VEGF, and procoagulant microparticles into the tumoral microenvironment (Figure 2) [61].

Recently, evidence of this thrombotic paradox has been demonstrated: a study published in 2021 has shown for the first time, in an original mouse model of OSCC, that there is no elevated systemic thrombosis risk [62]. Authors found that, in vitro, OSCC cells induce platelet aggregation due to high TF activity; they confirmed this in vivo by detecting platelet aggregates and fibrin deposits in histological sections of primary mouse tongue tumors [62]. In orthotopic animal models, there was no significant difference in terms of blood cell counts or tail bleeding times between healthy and cancer-inoculated mice [62]. Using the same OSCC orthotopic mouse model, researchers studied platelet accumulation and fibrin generation in the cremasteric arteries after a laser induced injury. Surprisingly, platelet accumulation and fibrin generation were highly inhibited in tumor-bearing mice versus healthy controls, suggesting decreased thrombotic power in OSCC [62].

Neutrophils are now known to be key players in both thrombosis and CAT [72]. More specifically, in this laser model of injury neutrophil they play a key role in thrombus initiation as they are the first cells detected at the endothelial injury site [72,73]. However, no difference in neutrophil accumulation at the site of injury was demonstrated, showing that the difference is due to a platelet defect and not a decrease in neutrophil activity [73].

Researchers determined that this discrepancy between in vivo and in vitro observations in OSCC was due to a decrease in αIIbβ3 activated form at the platelets’ surface, and a significant decrease in the number of alpha and dense granules present in the platelets of the cancer-bearing mice [62]. The authors show that locally, OSCC can cause multiple microthrombi and fibrin accumulation by aggregating platelets. Platelets are activated and aggregated in situ due to cancer-cell procoagulant protein expressions such as TF and thrombin. At the same time, OSCC has a low systemic risk of thrombus development because circulating platelets present a storage pool deficiency [61,62].

## 4. Colorectal Cancer

Colorectal cancer (CRC) affected 1.8 million people worldwide in 2017, of which 896,000 died [74]. This makes it the third most common cancer after lung cancer and breast cancer in women or prostate cancer in men [75]. Studies have also estimated that by 2035, the mortality of colorectal cancer will increase from 60 to 71.5% [76]. These data are partly explained by the fact that patients with CRC have abnormal activation of the coagulation cascade, which significantly increases the risk of VTE [5,77]. Therefore, there is a need to find treatments to reduce CAT, which results from the interaction of platelets with colorectal cancer cells.

Wu and colleagues demonstrated that the mean platelet volume (MPV) is elevated in several types of cancers, particularly in CRC [78]. This change in morphology reflects an increase in the production of platelets and consequently in the number of circulating platelets [78]. This may be because colorectal cancer cells are able to secrete IL-6 and stimulate platelet production by increasing thrombopoietin (TPO) secretion from hepatocytes (Figure 2) [79]. This phenomenon is generally a sign of advanced disease and of poor prognosis [77]. Indeed, colorectal cancer cells stimulate the production of platelets to maintain a normal number of platelets in the bloodstream because these are consumed during the formation of micro-thrombi [77,80,81]. This disruption of haemostasis facilitates tumor progression, angiogenesis, and metastasis by facilitating exchanges between cancer cells and platelets, and their arrest in the endothelial wall for extravasation [77,80,81].

Interestingly, the increase in MPV also highlights that massive platelet activation is taking place [78]. Mitrugno et al. demonstrated that colon cancer cells are able to activate platelets by expressing TF and generating thrombin, thus activating platelet receptor PAR4 and releasing both ADP and TXA2 into the tumoral microenvironment [82]. This platelet activation leads to fibrin generation and to increased thrombotic risk.

Moreover, several studies have shown that the daily use of low-dose anti-platelet drugs such as aspirin could prevent the development of metastasis and have a long-term chemo-preventive effect on colorectal cancer development [83,84,85]. These effects can be explained by the fact that aspirin targets platelet cyclooxygenase 1 (COX-1) [86]. Indeed, it is able to donate its acetyl group to acetylate a hydroxyl group present at the active site of COX-1, and as result there is an irreversible inhibition of the enzyme by steric blockage, preventing further platelet COX-1 and, eventually, TXA2 production, which reduces platelet aggregation [86]. Aspirin also inhibits cancer-induced inflammation by targeting the cyclooxygenase COX-1 and COX-2 because these enzymes lead to the production of prostaglandins, and it is known that cytokines plays an essential role in inflammation and thus in colorectal tumor development [87].

The interaction between platelets and colorectal cancer cells also induces the secretion of platelet-derived, pro-coagulant extracellular vesicles that play a role in CAT [82]. Colorectal cancer cells and platelets are in constant interaction in the tumoral microenvironment. Cancers cells interacting with platelets initiate tumoral EMT, and they also potentiate metastatic cells’ interplay with endothelial cells in a TCIPA-independent manner [40]. Some studies have described the presence of chimeric microparticles that contain both platelet and cancer protein-markers in their membrane, for example, PECAM-1 and CD41 [40]. In the microenvironment, these chimeric MVs are responsible for the monocyte’s recruitment at the primary tumor site. Moreover, they contain two pro cytokines (IFNγ and IL4) known to be implicated in the activation of tumoricidal function of macrophages. In the blood compartment, the same chimeric MVs prime the endothelium to enhance the adhesion of tumor cells by increasing their ICAM-1 expression [40].

The study of D. Mege and collaborators show that the microparticulosome signature is specific to colorectal cancer and is different from benign inflammatory colorectal disease [88]. Additionally, when patients entered remission the microparticulosome signature changed towards the signature seen in healthy control patients [88]. The researchers conclude that the microparticulosome and, more specifically, fibrin-bearing MVs can be used as a complex biomarker for both cancer progression and thrombotic risk [54,88,89]. In 2016, Zhao and colleagues highlighted that a high level of phosphatidylserine on the surface of platelets and their microparticles is associated with increased pro-coagulant activity in vitro that, moreover, corresponds to the different stages of CRC [90]. Taken together, these data demonstrate how platelet and cancer-cell interactions; platelet activation; and MV generation contribute to the pro-thrombotic phenotype of patients with CRC [82].

Colorectal cancer is a complex disease that moderately increases systemic thrombotic risk compared to the healthy population. This increase in risk is due to different types of interactions between cancer cells and platelets. Indeed, both TCIPA and TCIPA-independent mechanisms come into play, and, depending, on the receptors expressed by cancer cells, these interactions result in specific changes in properties of both tumor-cells and platelets that affect the overall organism [40,82].

## 5. Pancreatic Cancer

Pancreatic cancer is the fourth most deadly cancer in the world, with a median survival after diagnosis of 17–23 months. It is a cancer type that is more prevalent in developed countries, and its incidence increases steadily every year [91]. Pancreatic ductal adenocarcinoma (PDAC) compromises the majority of the histological types and is particularly aggressive, with a 5-year overall survival rate of 10% [92]. PDAC is considered one the most prothrombotic cancer types and interacts in direct and indirect manners with platelets to induce their activation, degranulation, and aggregation, which support tumor growth and spread [3,22,51].

PDAC cells are known to express thrombin on their surface, which directly engage with the PAR receptors on platelets leading to their activation [22,41,93]. In an indirect manner, thrombin can also engage with coagulation factors V, VIII, XI, and XIII, leading to activation of the coagulation cascade and the development of CAT [22]. Pancreatic adenocarcinoma cells can also express TF, the main activator of the coagulation cascade once it interacts with factor VIIa, and can thus further stimulate the development of thrombotic events [22,55]. Indeed, PDAC cells secrete platelet agonist into their microenvironment in a bid to increase TCIPA and local inflammation. It has been recently confirmed that both mouse and human PDAC cells secrete higher amounts of ADP into the tumoral microenvironment than washed platelets [51]. This ADP activates P2RY1 and P2RY12 in platelets to induce TCIPA and platelet degranulation in the tumoral microenvironment [51]. Recently, Palacios and collaborators demonstrated an increase of P2Y12 expression in tumoral pancreatic cancer tissue; moreover, the inhibition of P2RY12 using clopidogrel in pancreatic cancer not only reduces TCIPA but also tumor growth, metastasis, and the spontaneous thrombosis associated with Trousseaus syndrome [51]. These results showcase the powerful effect of platelets in malignant disease progression (Figure 2) [51,56].

In an indirect manner of interaction, PDAC cells are known for producing large amounts of MVs, which contain the same procoagulant proteins as the original cancer-cells on their membrane [22,24,54,88,94,95,96]. MVs with membrane-bound TF accumulate at thrombotic sites in an orthotopic pancreatic cancer mouse model, and their presence drastically increases the thrombotic risk [24,56]. TF bearing MVs also have integrins on their surface, allowing them to interact and bind to platelets in both the tumoral microenvironment and circulation [24,96]. This allows metastatic cancer cells to bind to circulating platelets to use as a shield against shear forces and the immune system [22].

In another study, PDAC-derived MVs were found to express fibrin, the final molecule in the coagulation cascade and that is issued after platelet–cancer-cell interactions [54]. Remarkably, these fibrin-bearing MVs could have potential as a biomarker for CAT development risk [54]. Authors isolated and studied mRNAs present in the fibrin bearing MVs and detected transcripts of five genes: ITGB1, ANXA1, ANXA5, HPGD, and NFKB1 [54]. ITGB1, also called integrin β1, is implicated in metastatic diffusion [54]. It has been shown that overexpression of ITGB1 may be a marker of poor prognosis in patients with PDAC who have undergone resection [97]. ANXA1 and ANXA5 encode annexin 1 and 5, respectively, which are calcium-dependent phospholipid-binding proteins involved in particular in drug resistance and coagulation [54,97]. Finally, HPGD and NFKB1 are involved in inflammation [54]. In fact, HPGD catalyzes the first step of the catabolic pathway of prostaglandins, cytokines involved in inflammation, and NFKB1 is a key transcription factor that stimulates the expression of genes involved in various biological functions. Inappropriate inactivation of NFKB1 is associated with various inflammatory diseases, including cancer [98]. All these proteins are therefore important for different stages of tumor progression, and their presence in the fibrin-bearing MVs attests to the power these entities have in cell–cell communication and in cancer growth and spread.

PDAC-secreted MVs are intricately related to cancer cells and their interactions with the microenvironment, so much so that their use as biomarkers for disease progression has been proposed. Indeed, D. Mege et al. have shown that the “microparticulosome” signature is different in cancer vs. benign disease and reverts to “healthy” parameters when disease is resolved in both colorectal and pancreatic afflictions [88].

As can be evidenced, pancreatic cancer in general and PDAC specifically can directly and indirectly interact with platelets to generate “tumor-educated” platelets and “platelet-educated” tumor cells that increase cancer aggressiveness and augment the risk of developing thromboembolic events [2,22,36,98]. Pancreatic cancer is renowned for its high thrombotic risk, and while we have focused on the interactions that involve platelets, there are other pathways that increase cell adhesion and encourage metastasis by hijacking other elements of the coagulation cascade; however, they surpass the scope of this review and have already been summarized elsewhere [2,22,36,98].

## 6. Conclusions

Trousseau’s syndrome is an important contributor to morbidity and mortality in the cancer setting. Traditionally, cancer-cell–platelet interactions have taken the spotlight to explain the increase in thrombotic risk in cancer patients. Here, we have strived to highlight the differences in platelet–cancer interactions and their consequences in the thrombotic risk across three different cancer types. As the evidence shows, all three types interacted directly and indirectly with the platelets, but the systemic risk in CAT is very different. This shows that each cancer type, and its interactions within the cancer microenvironment, are unique and therefore have unique effects on tumor growth, TCIPA, and thrombotic risk. It is important to understand the different mechanisms involved in cancer-associated thrombosis so that patients can be treated with the appropriate anti-coagulant or antiplatelet agents.

## Abbreviations

ADAM9	ADAM Metallopeptidase Domain 9
ADP	Adenosine Diphosphate
ALK	Anaplastic Lymphoma Kinase
ANXA1/5	Annexin A1/5
BRCA2	Breast cancer type 2 susceptibility protein
CAT	Cancer-associated thrombosis
CDKN2B	Cyclin-dependent kinase 4 inhibitor B
CLEC-2	C-type lectin-like type II transmembrane receptor
COX-1/2	Cyclooxygenase 1/2
CRC	Colorectal cancer
CTNNB1	Catenin beta-1
GPIbα	Glycoprotein Ib alpha
GPIV	Glycoprotein IV
HNC	Head-and-neck cancer
HPGD	15-hydroxyprostaglandin dehydrogenase (NAD(+))
ICAM1	Intercellular adhesion molecule 1
IDH1/2	Isocitrate dehydrogenase (NADP) cytoplasmic or mitochondrial
IL1/6	Interleukin 1/6
ITGB1	Integrin beta-1
KEAP1	Kelch-like ECH-associated protein 1
KRAS	Proto-Ongene GTPase
MET	Hepatocyte growth factor receptor
MPV	Mean platelet volume
MVs	Microvesicles
NFKB1	Nuclear factor NF-kappa-B p105 subunit
OSCC	Oral squamous cell carcinoma
P2RY1/P2RY12	Purinergic Receptor P2Y1/P2Y12
PAR	Protease-Activated Receptors
PDAC	Pancreatic Ductal Adenocarcinoma
PLF4	Platelet factor 4
PSGL-1	P-selectin glycoprotein ligand-1
ROS	Proto-Ongene 1, Receptor Tyrosine Kinase
STK11	Serine/threonine-protein kinase STK11
TCIPA	Tumor-cell-induced-platelet-aggregation
TF	Tissue Factor
TNFα	Tumor Necrosis Factor α
TPO	Thrombopoietin
TXA2	Thromboxan A2
VEGF	Vascular Endothelial Growth Factor
VTE	Venous thromboembolism
vWF	Von Willebrand Factor
